# Insight into Antifungal Metabolites from *Bacillus stercoris* 92p Against Banana Cordana Leaf Spot Caused by *Neocordana musae*

**DOI:** 10.3390/biom14121495

**Published:** 2024-11-24

**Authors:** Qunfang Yu, Pengbo He, Yanxiang Qi, Pengfei He, Ayesha Ahmed, Xin Zhang, He Zhang, Yixin Wu, Shahzad Munir, Yueqiu He

**Affiliations:** 1 State Key Laboratory for Conservation and Utilization of Bio-Resources in Yunnan, Yunnan Agricultural University, Kunming 650201, China; 2 Institute of Environment and Plant Protection, Chinese Academy of Tropical Agricultural Sciences, Haikou 570100, China

**Keywords:** cordana leaf spot of banana, *Neocordana musae*, *Bacillus stercoris*, antifungal activity, lipopeptide

## Abstract

Banana crop ranks among the most crucial fruit and food crops in tropical and subtropical areas. Despite advancements in production technology, diseases such as cordana leaf spot, caused by *Neocordana musae*, remain a significant challenge, reducing productivity and quality. Traditional chemical controls are becoming less effective due to the development of resistance in target pathogens, which pose significant environmental and health concerns. Consequently, there is growing attention toward the development of biocontrol strategies. Here, we identified a new bacterial strain, *Bacillus stercoris* 92p, from the rhizosphere soil of banana. We evaluated its ability to suppress the growth of *N. musae* and other fungal pathogens that cause leaf spot disease in bananas. The inhibitory effect of *B. stercoris* 92p were checked using dual culture assays, microscopic observations, and pot experiments. Furthermore, the biocontrol mechanisms were investigated using whole-genome sequencing and biochemical analyses. The results showed that *B. stercoris* 92p exhibited significant antifungal activity against *N. musae* and other fungal pathogens, with inhibition rates exceeding 70%. Microscopic examination revealed significant morphological alterations in the hyphae and conidia of the tested pathogens. In pot experiments, *B. stercoris* 92p effectively reduced the severity of cordana leaf spot, achieving a biocontrol efficacy of 61.55%. Genomic analysis and biochemical tests indicated that *B. stercoris* 92p produces various antifungal compounds, including lipopeptides (fengycins and surfactins), hydrolytic enzymes (proteases and amylases), and phosphate-solubilizing metabolites. In conclusion, the study highlights that *B. stercoris* could potentially be used as a potential biological control agent against cordana leaf spot.

## 1. Introduction

Banana is an important fruit crop in the tropical and subtropical regions of the world, serving as a primary food source and a significant economic commodity. Despite its long-standing agricultural importance, banana productivity and quality are continually challenged by persistent banana diseases, which remain a primary constraint [1,2]. Cordana leaf spot (CLS), caused by the fungal pathogen *Neocordana musae*, is a widespread foliar disease affecting banana cultivation, posing a substantial threat to plant health and productivity across the world [3]. The disease primarily manifests as large necrotic lesions on banana leaves, characterized by light brown, elliptical to fusiform shapes with distinct light grey centers and concentric ring patterns. Severe infections can lead to dry leaves and serious defoliation of plantains, thus affecting photosynthesis and yield [4,5,6]. Chemical control is the most common management approach for banana CLS [7]. However, long-term use of this method has limitations, including an evolution of pathogen resistance and significant risks to human health, food safety, and environmental integrity. Therefore, the development of biological control strategies has been receiving increased attention. The species of *Bacillus* are widely applied as biological control agents to combat various economically significant plant pathogens, including *Magnaporthe oryzae*, *Botrytis cinerea*, *Fusarium graminearum*, *F. oxysporum*, and *Colletotrichum* spp. [8,9,10,11,12,13,14]. *Bacillus* species exert their biocontrol effects primarily through three mechanisms: (1) colonizing plant and competing with pathogens for ecological niches and nutrients, (2) producing various antimicrobial metabolites that directly suppress the growth of phytopathogens and (3) induced systemic resistance (ISR) in plants. Among these mechanisms, the production of antifungal compounds stands as the primary strategy employed by microbial antagonists to suppress phytopathogens. For instance, *B. velezensis* FZB42 is known to produce bacillomycin D, surfactin, and fengycin and has been reported to effectively suppress the growth of various plant pathogens, such as *F*. *graminearum, Rhizoctonia solani,* and *Botrytis cinerea* [15,16]. Moreover, *B. amyloliquefaciens* DHA6 has been reported to produce iturin, surfactin, bacillomycin, syringfactin, and pumilacidin, which exhibited significant antifungal activity against *F. oxysporum* f. sp. *niveum* by disrupting structural integrity, inhibiting mycelial growth and spore germination. Despite extensive research on the application of *Bacillus* species for plant disease management, there is a paucity of literature investigating the biocontrol efficacy of these bacteria against CLS disease in bananas.

*B. stercoris* was isolated from a bioreactor used for processing commercial food waste in Australia in 2016. Initially identified as *Bacillus subtilis* subsp. *stercoris* based on an average nucleotide identity of 95.6% between the genomes of *B. subtilis* subsp. *subtilis* and *B. subtilis* subsp. *stercoris.* In 2020, *B. stercoris* was reclassified as a distinct species [17]. Previous reports indicated that *B. stercoris* exhibits significant biocontrol characteristics. Wang et al. isolated *B. stercoris* LJBS06 from the rhizosphere soil of grapevines, and it demonstrated broad-spectrum antifungal activity against multiple phytopathogens [18]. Moreover, Rattana et al. found that *B. stercoris* B.PNR1 has shown promising results in controlling the Fusarium wilt of tomato by inducing abnormal fungal cell wall structures and causing the cell wall to collapse. Both *B. stercoris* strains B.PNR1 and LJBS06 were reported to harbor many genes responsible for secondary metabolite production, although their specific identification remains unknown [19]. These findings suggest that *B. stercoris* has the potential to effectively inhibit the growth of multiple plant pathogens, positioning it as a promising candidate for use as a biocontrol agent in sustainable agricultural practices.

The primary aims of the current research were (1) to isolate, screen, and identify bacterial strains performing antifungal activity against *N. musae*, (2) to evaluate the biocontrol efficiency of *B. stercoris* 92p in managing CLS in bananas, and (3) to investigate the specific biocontrol mechanism of *B. stercoris* 92p against *N. musae*. To our knowledge, this is the first study to report how the *B. stercoris* strain may effectively control CLS in bananas. The findings of our study suggest that *B. stercoris* is a promising option for future development and application as a control measure for CLS in banana crops.

## 2. Materials and Methods

### 2.1. Pathogen Strains and Plant Material

The pathogens: *Neocordana musae* (MW185976.1), *Corynespora torulosa* (MZ736144.1), *Exserohilum rostratum* (GQ169762.1), *Phyllosticta capitalensis* (MW412576.1), *Curvularia lunata* (MW186186.1), *Neopestalotiopsis clavispora* (OM281005.1), and *Nigrospora sphaerica* (MW186164.1) previously isolated from banana leaves were cultured on potato dextrose agar (PDA). The banana cultivar ’Brazilian’ (*Musa acuminata* L. AAA group) was used as an experimental plant in the pot experiments.

### 2.2. Isolation and Purification of Bacteria from Banana Rhizosphere Soil

Soil samples were obtained from the rhizosphere of banana plants in Hainan Province, China [20]. Five grams of each soil sample were placed into an Erlenmeyer flask (100 mL) containing ten sterile 5-mm glass beads and 50 mL of sterile deionized water. The flasks were then agitated at 37 °C and 200 rpm for 60 min. Sequential ten-fold dilutions of the soil suspensions were prepared in sterile water. Aliquots (100 μL) of the properly diluted suspensions were inoculated onto Luria Bertani (LB) agar plates using the spread-plate technique. Subsequently, the plates were incubated for two to three days at 37 °C. Following incubation, colonies exhibiting distinct morphological characteristics were selected and purified using the successive streak plate method. These purified single colonies were inoculated into LB broth and cultured under orbital agitation at 200 rpm and 37 °C for 24 h. The bacterial cultures were preserved at −80 °C in 20% glycerol for future use.

### 2.3. In Vitro Antagonism Assay for Bacterial Isolates Against N. musae

The antifungal potential of the isolated bacterial strains was evaluated through a dual culture technique, with *N. musae* as the target fungal pathogen [21]. *N. musae* was cultured on PDA plates for 10 days, and 8-mm mycelium plugs were excised and centrally placed on fresh PDA plates. Subsequently, 5 μL portions of bacterial culture were placed on the agar surface at four evenly spaced spots. Each inoculation point was positioned 2.0 cm away from the center of the fungal mycelial plug. PDA plates solely inoculated with *N. musae* were used as the control in the experiment. All plates were incubated at 28 °C for 10 days, and the growth of *N. musae* colonies was recorded by measuring their diameter. The percentage of inhibition (I%) was calculated using the following formula: I% = [(C − T)/C] × 100.

Where: “C” represents the diameter of the *N. musae* colony on control plates (without bacterial inoculation), and “T” indicates the diameter of the *N. musae* colony on dual culture plates (with bacterial inoculation).

### 2.4. Identification of Strain 92p

The isolate 92p was identified using molecular sequencing of the *16S rRNA* and *gyrB* genes, followed by phylogenetic analysis. Genomic DNA extraction for isolate 92p was performed using the Aidlab DNA extraction kit (Aidlab Biotechnologies Co., Ltd., Beijing, China). PCR amplification of the *16S rRNA* gene and *gyrB* gene was carried out according to the protocol described in the literature [22,23]. The PCR products were sequenced and subjected to phylogenetic tree construction using MEGA 6 software, employing the maximum likelihood method with a bootstrap of 1000 replications.

### 2.5. Antifungal Spectrum Tests

Using the dual culture approach mentioned above, the antifungal spectrum of Strain 92p was determined against six fungal pathogens. The tested pathogens included *Cor. torulosa,* which causes banana helminthosporium leaf spot, *E. rostratum* causes exserohilum leaf spot, *P. capitalensis* responsible for guignardia leaf spot, *Cur. lunata* causes curvularia leaf spot, *Neo. clavispora* causes leaf spot, and *Nig. sphaerica* causes nigrospora leaf spot in bananas. PDA plates inoculated solely with the fungal pathogen served as the negative control group. To evaluate the inhibitory effect of Strain 92p on the hyphal morphology of each pathogenic fungus, microscopic observations were performed using an optical microscope, the Nikon ECLIPSE Ni upright microscope (Nikon, Tokyo, Japan).

### 2.6. The Impact of B. stercoris 92p Cell-Free Supernatant on the Growth of N. musae Hyphae

*B. stercoris* 92p was cultured in potato dextrose broth (PDB) and incubated at 37 °C with constant agitation at 200 rpm for 48 h. The bacterial culture underwent centrifugation at 4 °C for 10 min at 12,000 rpm. To ensure sterility, the resulting supernatant was filtered through a 0.22 μm sterile membrane. The sterilized supernatant of *B. stercoris* 92p was mixed with PDA to final concentrations of 10%, 20%, and 30%. A mycelium plug (8 mm in diameter) excised from a two-week-old culture of *N. musae* was placed at the central point of the prepared PDA medium [24], and the experiment was conducted in triplicate. PDA plates without *B. stercoris* 92p culture filtrate served as the control group.

### 2.7. Control Efficacy of B. stercoris 92p Against Cordana Leaf Spot of Banana

To assess the biocontrol efficacy of Strain 92p against CLS of banana, six mycelium blocks were inoculated on each banana leaf using the pin-prick method. Three days after inoculation with *N. musae*, the banana plants were sprayed with a suspension of 92p (10^8^ CFU/mL) containing 0.02% Tween 80. The negative control group was treated with sterile water containing 0.02% Tween 80 and 80% carbendazim wettable powder (WP) at a concentration of 1 mg/L, supplemented with 0.02% Tween 80 served as the positive control. Fourteen days post-inoculation, the diameters of the lesions were measured, and the control efficacy was recorded using the following formula: Control efficacy (%) = [(Lesion area in untreated plants − Lesion area in treated plants)/Lesion area in untreated plants] × 100.

### 2.8. In Vitro Assessment of Biocontrol and Plant Growth-Promoting Characteristics of B. stercoris 92p

Carboxymethyl cellulose (CMC) agar, skimmed milk medium, soluble starch agar medium, and β-1,3-Glucanase medium were used to evaluate the enzymatic activity of *B. stercoris* 92p, including cellulase, protease, amylase, and β-1,3-glucanase productions. The pikovskava (PVK) medium was employed to assess phosphate solubilization, and the Chrome Azurol-S (CAS) detection medium was used for siderophore production. *B. stercoris* 92p was inoculated to all of these media plates and then incubated for three to seven days at 37 °C. The presence of a hydrolytic halo suggested the ability to produce enzymes and solubilize phosphate. In contrast, a yellow-orange halo on the CAS medium served as an indicator of siderophore production.

### 2.9. Complete Genome Sequencing and Analysis of B. stercoris 92p

*B. stercoris* 92p was subjected to genome sequencing using the Illumina NovaSeq 6000 and PacBio Sequel II technologies at Shanghai Winnerbio Technology Co., Ltd. (Shanghai, China). The sequencing data were assembled using the Canu v2.2 and Pilon v1.24. Gene prediction was performed using prodigal v2.6.3 with default parameters. RNAmmer v1.2 and tRNAscan-SE v2.0.8 were used to identify tRNA and rRNA. The predicted genes were annotated by searching against several databases: Non-Redundant (NR) Protein, Swiss-Prot, protein families (Pfam), Clusters of Orthologous Groups (COG), Gene Ontology (GO), and Kyoto Encyclopedia of Genes and Genomes (KEGG). The antiSMASH platform v6.0.0 was employed to predict putative gene clusters involved in the biosynthesis of the secondary metabolite of Strain 92p. Then, we performed average nucleotide identity (ANI) analysis using FastANI v1.34 with default parameters, using the nucleotide sequences directly retrieved from NCBI [25]. The online tool ChiPlot (https://www.chiplot.online/, accessed on 25 December 2023) was selected to generate a heatmap visualization.

### 2.10. Extraction, Purification, and Characterization of Extracellular Lipopeptides from B. stercoris 92p

The extract of lipopeptides was obtained using a method described previously [26] with minor adjustments. Bacterial Strain 92p was cultured in 20 mL of LB at 37 °C for 24 h with agitation at 180 rpm. A 10 mL culture of 92p was added to 200 mL of Landy medium and incubated at 37 °C for three days with shaking at 180rpm. The supernatant was collected by centrifugation at 12,000 rpm for 10 min at 4 °C. Then, the supernatant was acidified to pH 2.0 using 6M HCl and kept at 4 °C overnight. The precipitates were collected by centrifugation at 4 °C for 10 min at a speed of 12,000 rpm and dissolved in methanol. The pH value of the methanolic solution was neutralized to 7.0 by using 1 M NaOH. The mixture was vortexed for two min, and the supernatant was evaporated using a rotary vacuum. Subsequently, the dried lipopeptides were re-suspended in methanol.

UHPLC-QTOF-MS/MS analysis was carried out to identify the antifungal compounds by utilizing a TripleTOF^TM^ 6600 (Sciex, Foster City, CA, USA) [27]. The analytes were separated chromatographically using a Kinetex XB-C18 column (2.1 mm×100 mm, 2.6 μm) (Phenomenex, Torrance, CA, USA). The two mobile phases for the chromatographic separation were HPLC grade 0.1% formic acid aqueous solution (A) and methanol (B). The program for optimized linear gradient elution was performed as 0–60 min, 60–90% B; 60–65 min, 60–90% B. A sample volume of 1 μL was injected into the system at a flow rate of 0.40 mL/min. The data were analyzed using Analyst TF 1.7. and PeakView™ 2.0 software for mass spectral data analysis and interpretation. Data acquisition for each individual run was done using information-dependent acquisition, with an *m*/*z* range of 50–2000 for both TOF MS and MS/MS.

### 2.11. Statistical Analysis

Data was analysed using IBM SPSS Statistics 25.0 software (IBM, Westchester County, NY, USA). A one-way analysis of variance (ANOVA) was conducted to assess the significance and contribution of the samples. Subsequently, Duncan’s multiple range test was applied, with the significance threshold set at *p* < 0.05. All treatments were performed in triplicate to ensure reproducibility of the results.

## 3. Results

### 3.1. Isolation and Screening of Antagonistic Bacteria Against N. musae

**Figure 1:**
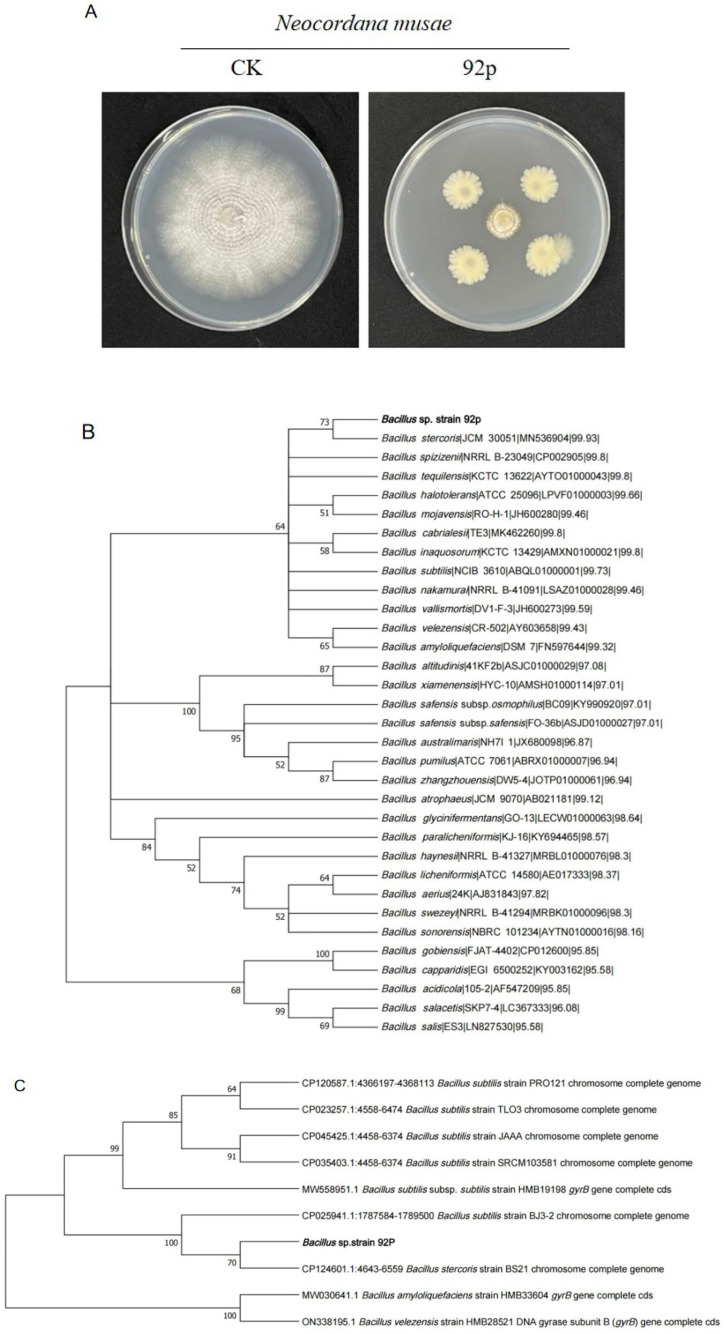
(**A**) Antagonistic activity of Strain 92p against *N. musae*. The phylogenetic tree of *Bacillus* sp. Strain 92p was constructed using the maximum-likelihood method for the analysis of two genes: the *16S rRNA* gene (**B**) and the *gyrB* gene sequence (**C**).

From the rhizosphere soil samples collected from banana plants, a total of 201 bacterial isolates were obtained. Screening of the strains revealed that eighteen strains exhibited antifungal activity against *N. musae*. The antagonistic bacterial isolates inhibited the mycelial growth of *N. musae* to varying extents, from 50.88 to 76.71% (Table S1). Among the 18 isolates, Strain 92p demonstrated the most potent antagonistic effects against *N. musae* with an inhibition rate of 76.71 ± 7.09% (Figure 1A).

### 3.2. Identification and Phylogenetic Analysis of Antagonistic Bacterium 92p

Morphological observations indicated that Strain 92p formed round, milky-white colonies with well-defined margins and a rough, opaque surface on LB agar (Supplementary Figure S1). Amplification and sequencing were performed with the *16S rRNA* and *gyrB* genes of Strain 92p, followed by phylogenetic analysis using the maximum-likelihood method. The results demonstrated Strain 92p clustered within the *B. stercoris* lineage (Figure 1B,C). Consequently, molecular and phylogenetic analyses confirmed Strain 92p as *B. stercoris*.

### 3.3. Antagonistic Activity of B. stercoris 92p Against Plant Fungal Pathogens

**Figure 2:**
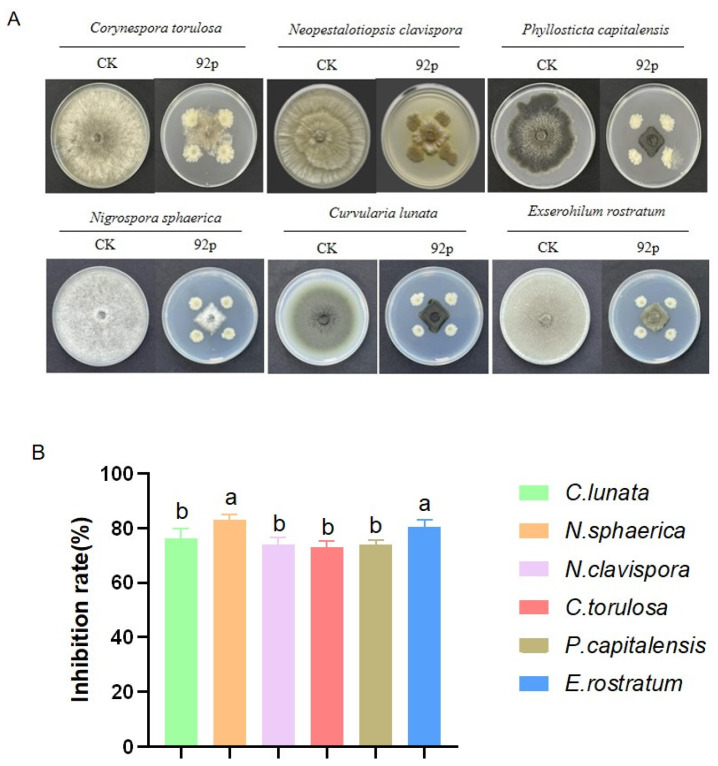
(**A**) Antagonistic activity of *B. stercoris* 92p against phytopathogenic fungi. Antifungal efficacy of *B. stercoris* 92p against phytopathogenic fungi was evaluated on PDA plates. (**B**) The graph illustrates the mycelial inhibition (%) by *B. stercoris* 92p on various phytopathogenic fungi. Bars marked with distinct letters exhibit statistically significant variations (*p* < 0.05) according to Duncan’s multiple comparison test.

The 92p strain demonstrated potent antifungal activity against multiple phytopathogens during in vitro experiments. The inhibitory effect of the 92p strain on *Cur. lunata*, *P. capitalensis*, *Cor. torulosa*, *Nigr. sphaerica*, *Neo. clavispora*, and *E. rostratum* exceeded 70%. The most pronounced antagonistic effects were observed against *Nigr. sphaerica*, the causal agent of spot disease in bananas, with an inhibition rate of 83.23 ± 1.77%, followed by *E. rostratum* (80.55 ± 2.36%) and *Cur. lunata* (76.71 ± 7.09%) (Figure 2A,B).

### 3.4. Microscopic Analysis of Fungal Pathogen Hyphae Post-Treatment

**Figure 3:**
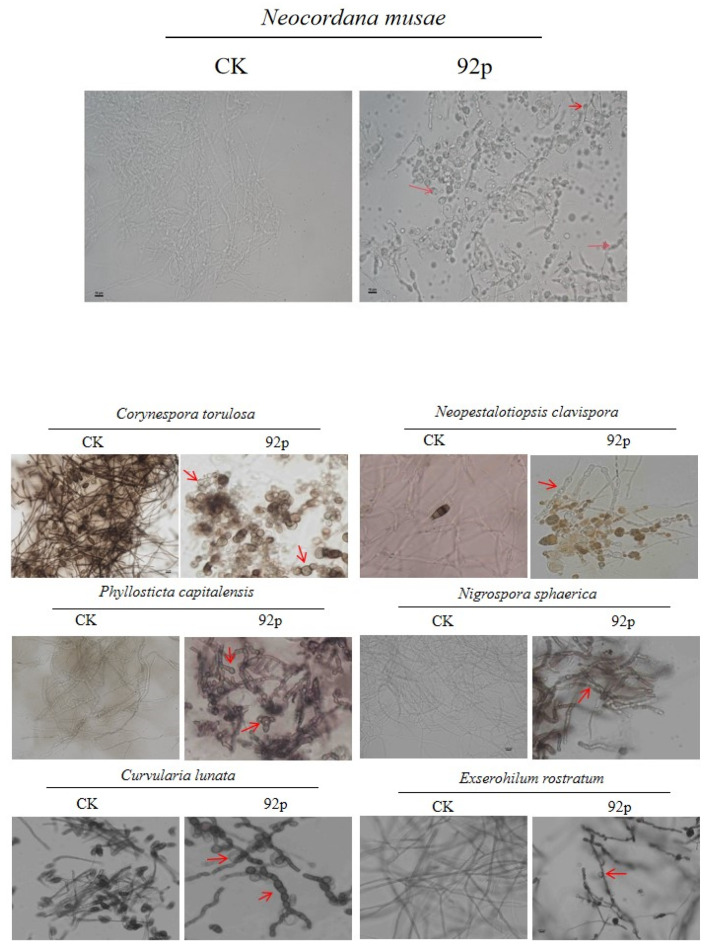
Morphological changes in fungal pathogen hyphae following treatment with *B. stercoris* 92p. Arrows highlight alterations, including distortions, dissolution, swelling, deformation, beadlike appearance, and vacuolation of structures induced by *B*. *stercoris* 92p. Scale bar: 10 μm.

Morphological changes induced by Strain 92p were observed using a microscope. Optical microscopic observations revealed that control mycelia and conidia appeared normal and smooth, whereas the samples treated with Strain 92p exhibited significant structural alterations. The hyphae of *P. capitalensis*, *Nigr. sphaerica*, *N. musae*, and *Neo. clavispora* subjected to inhibition by Strain 92p displayed severe deformities, distortions, swelling, and dissolution. Furthermore, the hyphae of *Cur. lunata* and *E. rostratum* treated with Strain 92p swelled and assumed a beadlike appearance at each internode. The conidia of *Cor. torulosa* and *Neo. clavispora* treated with Strain 92p underwent vacuolation (Figure 3).

### 3.5. Biocontrol Effects of Strain B. stercoris 92p Against Leaf Spot of Banana

**Figure 4:**
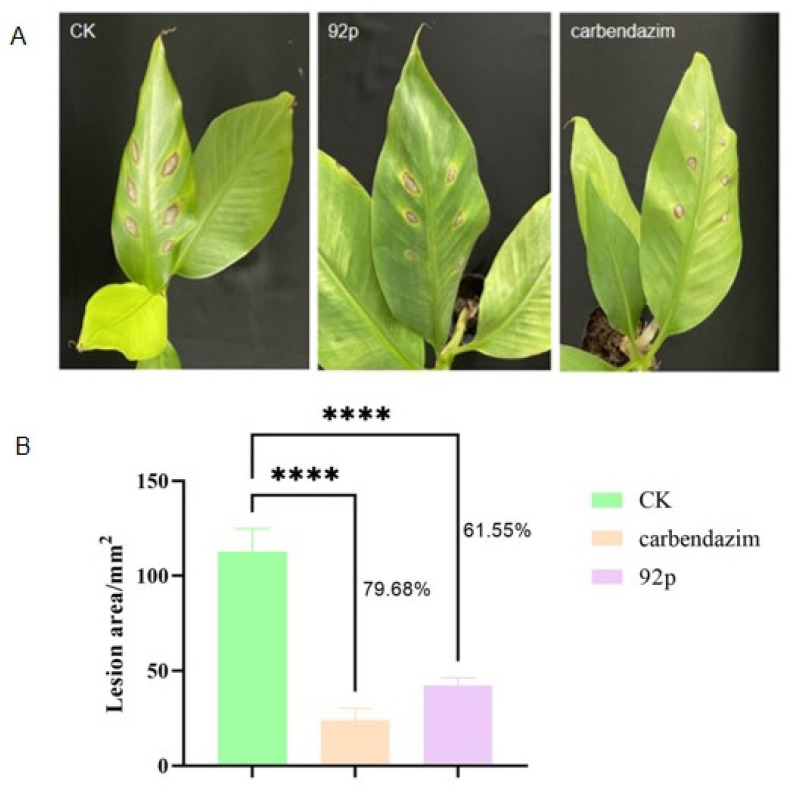
Biocontrol effects of *B. stercoris* 92p against cordana leaf spot of banana. (**A**) Banana leaves were treated with sterile water, Strain 92p, and carbendazim, respectively. (**B**) The lesion area of banana leaves after treatment with ddH_2_O, Strain 92p, and carbendazim. Asterisks indicate statistically significant differences according to Duncan’s multiple comparison test (**** *p* ≤ 0.0001).

Biocontrol efficacy evaluation of Strain 92p against *N. musae* was performed through pot experiments. On the fourteenth day after infection, typical disease symptoms began to appear in the banana leaves. Plants treated with Strain 92p or carbendazim exhibited less severe disease symptoms compared to control plants. The lesion area of disease spots in untreated leaves was 112.70 ± 11.95 mm^2^, while the plants treated with Strain 92p and carbendazim were 43.33 ± 6.81 mm^2^ and 22.90 ± 6.23 mm^2^, and the biocontrol efficacy reached 61.55% and 79.68%, respectively. These results indicate that Strain 92p had biocontrol potential against banana CLS (Figure 4).

### 3.6. Impact of the Cell-Free Supernatant from Strain 92p on the Mycelial Growth of N. musae

**Figure 5:**
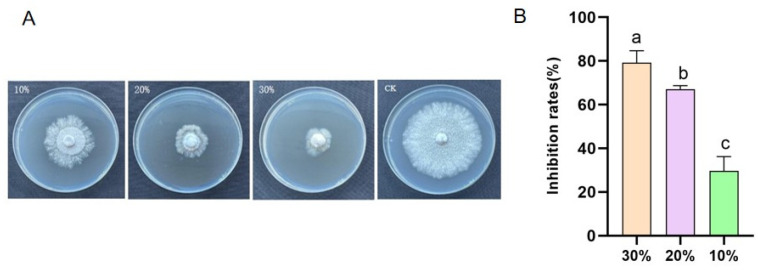
Inhibitory effect of *B. stercoris* 92p cell-free supernatant on *N. musae.* (**A**) PDA media were supplemented with varying concentrations (10%, 20%, and 30% *v*/*v*) of the 92p sterile supernatant and inoculated with *N. musae* mycelial discs. A non-supplemented PDA plate served as the control. (**B**) Bars marked with distinct letters exhibit statistically significant variations (*p* ≤ 0.05) according to Duncan’s multiple comparison test.

The mycelial growth of *N. musae* was significantly inhibited by the supernatant of the 92p strain at various concentrations. The PDA plates supplemented with 10%, 20%, and 30% (*v*/*v*) of the 92p fermentation filtrate exhibited inhibition rates of 29.63%, 67.13%, and 79.17%, respectively, compared to the control (Figure 5). The results demonstrate a dose-dependent suppression of *N. musae* mycelial growth by the 92p cell-free supernatant, suggesting the presence of antifungal compounds in the fermentation broth.

### 3.7. Analysis of Biocontrol Activities and Plant Growth-Promoting Traits of B. stercoris 92p

**Figure 6:**
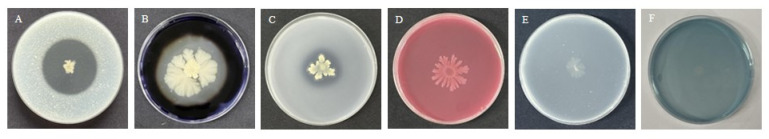
The enzymatic activity and the secondary metabolites produced by *B. stercoris* Strain 92p. (**A**) Protease production, (**B**) Amylase production, (**C**) Phosphate solubilization assay, (**D**) Cellulase production, (**E**) β-1,3-Glucanase production, (**F**) Siderophore production.

The results revealed that *B. stercoris* 92p could secrete amylase and protease and solubilize phosphorus, as evidenced by hydrolysis zones in skim milk, soluble starch, and phosphate solubilization media. The lack of transparent halos on cellulose and β-1,3-glucanase plates indicated that Strain 92p could not produce cellulase and β-1,3-glucanase enzymes. When inoculated on the CAS plate, Strain 92p did not exhibit a yellow halo, indicating its inability to produce siderophores (Figure 6).

### 3.8. Genome Sequencing Analysis of B. stercoris 92p

**Figure 7:**
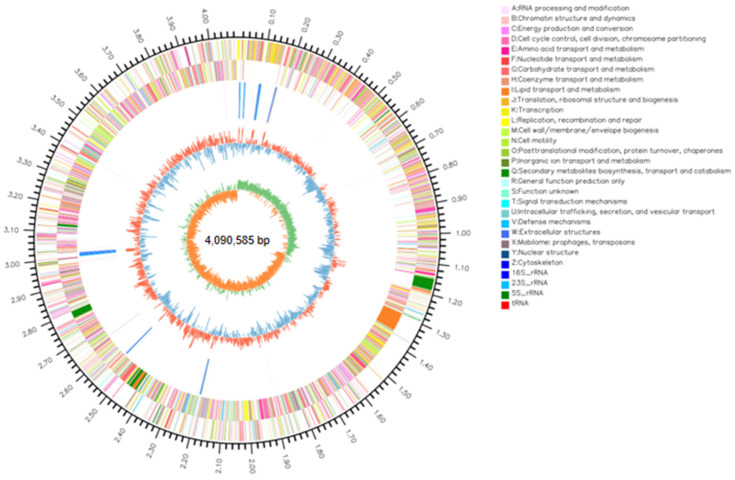
Circular map of *B. stercoris* 92p genome. The outermost circle visually represents the size and scale of the genome. The default circles from outer to inner correspond to genome size markers, gene information on the forward and reverse strand, non-coding RNA, repetitive elements, GC content, and GC-Skew.

**Figure 8:**
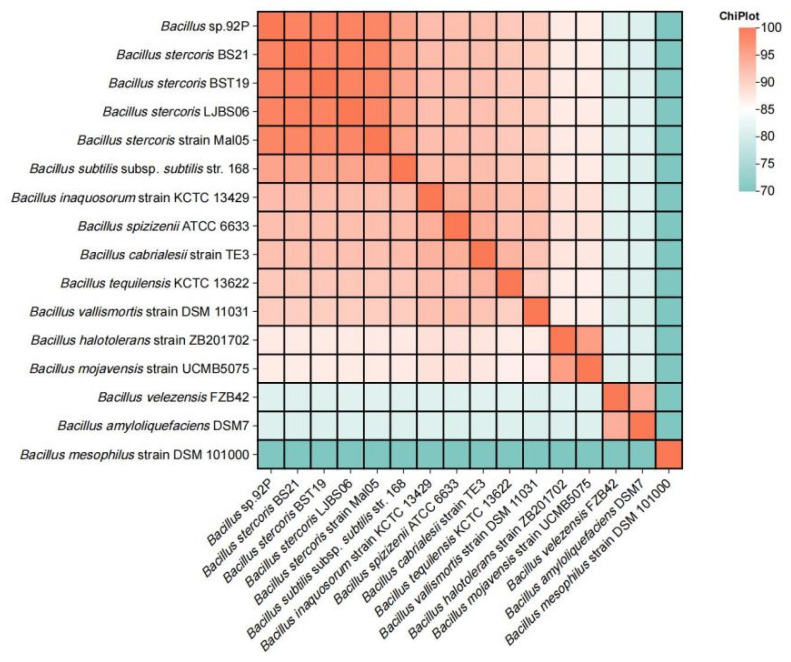
The heatmap displays the average nucleotide identity among strains 92p and 15 *Bacillus*. ANI values were computed using FastANI for pairwise genome comparisons, and the heatmap illustrates the percentage of ANI among 15 *Bacillus* strains, with higher values represented by reddish colors to distinguish strains of the same species.

The genome of *B. stercoris* 92p comprises a single circular chromosome spanning 4,090,585 base pairs with an average G + C content of 44.05%. Notably, no plasmids were detected in this strain’s genetic material. Genomic analysis revealed 3969 protein-coding sequences, along with 85 tRNA and 30 rRNA genes. The complete genomic sequence of *B. stercoris* 92p has been deposited in GenBank with accession number CP143103 (Figure 7). Initially, Strain 92p was determined to be *B. stercoris* by *16S rRNA* and *gyrB* gene sequencing and phylogenetic analysis. To further validate the classification, *B. stercoris* Strain 92p was compared to closely related *Bacillus* strains using Average Nucleotide Identity (ANI) analysis. The ANI analysis demonstrated that the genome sequence of Strain 92p exhibited the highest similarity to *B. stercoris*, with ANI values ranging from 98.50% to 98.80% (Figure 8), and classified Strain 92p as *B. stercoris*.

The COG annotation analysis of the 92p genome revealed that 3256 genes, representing 82.04% of the predicted gene complement, were successfully annotated using the COG database. These genes were classified into 23 distinct COG categories, with 17 categories encompassing over 100 annotated genes each (Supplementary Figure S2). The three categories with the largest protein-coding genes were amino acid transport and metabolism, followed by carbohydrate transport and metabolism and transcription, indicating a significant proportion of the genome is dedicated to these essential cellular processes.

In total, 1871 genes, representing 47.14% of the entire gene repertoire, were effectively annotated using the GO database (Supplementary Figure S3). Within the annotated MF category, genes involved in ATP binding, DNA binding, metal ion binding, and hydrolase activity were the most predominant functional groups, highlighting the importance of these molecular functions in the organism’s cellular processes. The KEGG pathway annotation of the genome revealed that 2458 genes, constituting 61.93% of the total encoded genes, were functionally annotated. These genes were categorized into six main biological metabolic pathways (Supplementary Figure S4), with metabolism being the most represented category (77.53%). Within the metabolism category, 727 genes were enriched in metabolic pathways, while 342 genes were associated with the biosynthesis of secondary metabolites, underscoring the organism’s significant metabolic capabilities.

Regarding hydrolase, we annotated the *amyA* and *epr* genes, which encode α-amylase and protease, respectively. This finding aligns with in vitro experiments showing that *B. stercoris* 92p can produce amylase and protease. The genome of *B. stercoris* 92p contains several genes related to phosphate metabolism: the phosphate-specific transport system gene cluster (*pstABCS*), the phosphate regulon gene cluster (*phoADEPRH*), and the pyrroloquinoline quinone gene cluster (*pqqCDE*). These genes and their products work together to sense, solubilize, transport, and regulate phosphate levels within the bacterial cell. This process is crucial for survival and growth in phosphate-limited environments. Additionally, genes involved in colonization, swarming, and biofilm formation, such as *spo0A*, *sacB*, and *swrA*, were identified. These genes may also contribute to the biocontrol mechanisms of Strain 92p.

### 3.9. Gene Clusters Responsible for the Biosynthesis of Secondary Metabolites in B. stercoris 92p

AntiSMASH analysis of the genome of Strain 92p revealed the presence of eleven gene clusters accountable for secondary metabolite biosynthetic (Table 1). These clusters were categorized into various types, including four Nonribosomal peptides synthetases (NRPS), two terpenes, one type I Polyketide synthetases (PKS), one type III PKS, a single sactipeptide gene cluster, and one rifampin resistance element (RRE)-containing gene cluster. Additionally, one gene cluster with an unknown function was identified. Further investigation showed that clusters 3, 4, 8, 9, and 10 exhibited 100% amino acid sequence identity to previously characterized gene clusters known to be involved in fengycin, bacillaene, bacillibactin, subtilosin A, and bacilysin biosynthesis, respectively. Gene cluster 7 exhibited an 82% similarity to a gene cluster encoding a surfactin synthetase, while gene cluster 6 displayed an 18% similarity to a zwittermicin A synthetase gene cluster. These findings provide valuable insights into the secondary metabolite production potential of Strain 92p, highlighting its capacity to synthesize a diverse array of bioactive compounds.

### 3.10. UHPLC–QTOF–MS/MS Analysis of Lipopeptides in Strain 92p

The lipopeptides produced by *B. stercoris* 92p were characterized using UHPLC–QTOF–MS/MS analysis. The total ion chromatography (TIC) results displayed two signal peaks, concentrated at 45–55 min and 56–63 min, respectively. The lipopeptides were identified as two distinct families: surfactin and fengycin (Supplementary Figure S5).

**Figure 9:**
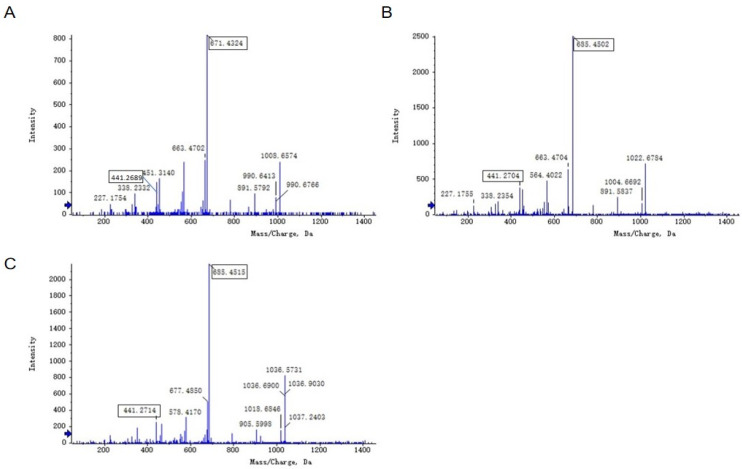
MS/MS spectra of surfactin ions. (**A**): *m*/*z* 1008.6574, (**B**): *m*/*z* 1022.6784, (**C**): *m*/*z* 1036.5731.

Surfactin exists in two primary forms: surfactin A, characterized by Leu at the 7th position of the cyclic lipopeptide, and surfactin B, which has Val at the identical place. Figure 9A displays the MS/MS spectrum of ion at *m*/*z* 1008. A series of fragments at *m*/*z* 891 (*β*-hydroxyl fatty acid-Glu^1^ -Leu^2^ -Leu^3^ -Val^4^ -Asp^5^ -Leu^6^), *m*/*z* 671 (Leu^2^ -Leu^3^ -Val^4^ -Asp^5^ -Leu^6^ -Val^7^), *m*/*z* 663 (*β*-OH fatty acid--Glu^1^ -Leu^2^ -Leu^3^ -Val^4^), *m*/*z* 441 (Leu^2^ -Leu^3^ -Val^4^ -Asp^5^) were found in the MS/MS spectrum. According to these typical fragments, the sequence could be deduced as a C14 *β*-hydroxyl fatty acid chain-Glu^1^ -Leu^2^ -Leu^3^ -Val^4^ -Asp^5^ -Leu^6^ -Val^7^, which was assigned as C14 surfactin B. Figure 9B displayed the MS/MS spectrum of the ion at *m*/*z* 1022. A series of fragments at 891 (*β*-hydroxyl fatty acid-Glu^1^ -Leu^2^ -Leu^3^ -Val^4^ -Asp^5^ -Leu^6^), *m*/*z* 685 (Leu^2^ -Leu^3^ -Val^4^ -Asp^5^ -Leu^6^ -Leu^7^), *m*/*z* 663 (*β*-OH fatty acid--Glu^1^ -Leu^2^ -Leu^3^ -Val^4^), *m*/*z* 441 (Leu^2^ -Leu^3^ -Val^4^ -Asp^5^), *m*/*z* 338 (*β*-OH fatty acid-Glu^1^) were found. Similarly, it was proposed that the precursor ion had a peptide sequence of Glu^1^ -Leu^2^ -Leu^3^-Leu^4^ -Asp^5^ -Leu^6^ -Leu^7^ and a C14 *β*-hydroxyl fatty acid chain, which was identified as C14 surfactin A. The MS/MS spectrum of ion at *m*/*z* 1036 also yielded similar fragments at *m*/*z* 685 (Leu^2^ -Leu^3^ -Val^4^ -Asp^5^ -Leu^6^ -Leu^7^) and *m*/*z* 441 (Leu^2^ -Leu^3^ -Val^4^ -Asp^5^) (Figure 9C), the sequence could be surmised as C15 *β*-OH fatty acid-Glu^1^ -Leu^2^ -Leu^3^ -Leu^4^ -Asp^5^ -Leu^6^ -Leu^7^. The *m*/*z* 1036 and *m*/*z* 1022 ions are homologs with the same amino acid sequence but different *β*-OH fatty acids. The data corresponded with the mass spectra of the surfactin standard (Rhawn, Shanghai, China) (Supplementary Figure S6) The surfactin homologs identified from *B. stercoris* 92p are presented in Table 2.

**Figure 10:**
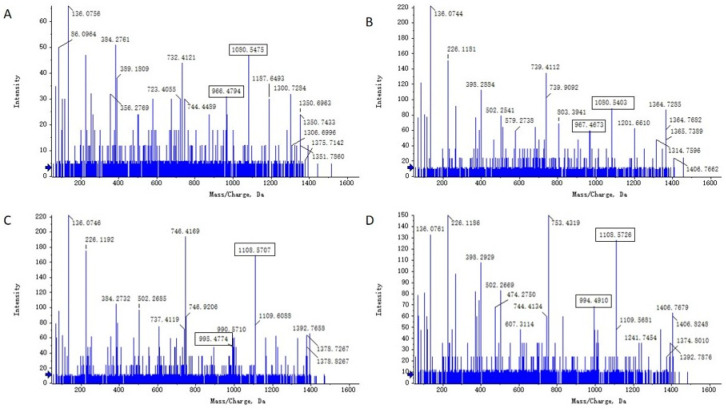
The MS/MS analysis of four fengycins (**A**–**D**). (**A**) *m*/*z* 1463.8242, (**B**) *m*/*z* 1477.8224, (**C**): *m*/*z* 1491.8338, (**D**) *m*/*z* 1505.8638. The black box labeled fingerprint product ions 1080 and 966 indicated that the *m*/*z* values 1463.8242 and 1477.8224 correspond to fengycin A. The black box labeled fingerprint product ions 1108 and 994 indicated that the *m*/*z* values 1491.8338 and 1505.8638 correspond to fengycin B.

Fengycin homologs are categorized into two distinct types: fengycin A and B, based on their amino acid sequences. Fengycin A has Ala at position 6, whereas fengycin B contains Val at the same position. Through collision-induced dissociation, the peptide chain of fengycin can generate some characteristic fragments, which can be used as fingerprints to identify the species of fengycin in various research. The ion peaks at *m*/*z* 1080 (Orn^2^ -Tyr^3^ -Thr^4^ -Glu^5^ -Ala^6^ -Pro^7^ -Gln^8^ -Tyr^9^ -Ile^10^) and 966 (Tyr^3^ -Thr^4^ -Glu^5^ -Ala^6^ -Pro^7^ -Gln^8^ -Tyr^9^ -Ile^10^) in secondary mass spectrometry are indicative of fengycin A, and the ion peaks at *m*/*z* 1108 (Orn^2^ -Tyr^3^ -Thr^4^ -Glu^5^ -Val^6^ -Pro^7^ -Gln^8^ -Tyr^9^ -Ile^10^) and 994 (Tyr^3^ -Thr^4^ -Glu^5^ -Val^6^ -Pro^7^ -Gln^8^ -Tyr^9^ -Ile^10^) are the characteristic ion peaks of fengycin B. Figure 10A,B showed two fingerprint product ions at *m*/*z* 1080 and 966, the sequence of *m*/*z* 1463.8242 and 1477.8224 could be concluded as Glu^1^ -Orn^2^ -Tyr^3^ -Thr^4^ -Glu^5^ -Ala^6^ -Pro^7^ -Gln^8^ -Tyr^9^ -Ile^10^ with a C16-C17 *β*- OH fatty acid, which was identified as C16-C17 fengycin A. The MS/MS spectrum of ions at *m*/*z* 1491.8338 and 1505.8638 yielded two fingerprint product ions at *m*/*z* 1108 and 994, which were identified as fengycin B with sequence Glu^1^ -Orn^2^ -Tyr^3^ -Thr^4^ -Glu^5^ -Val^6^ -Pro^7^ -Gln^8^ -Tyr^9^ -Ile^10^ and a C16-C17 *β*- OH fatty acid. The fengycin homologs identified from *B. stercoris* 92p are presented in Table 2 [26,27,28].

## 4. Discussion

In the present study, we identified a novel *Bacillus* Strain 92p from the rhizosphere soil of the banana plant. The taxonomic identity of the strain was determined to be *B. stercoris* based on *16S rRNA* and *gyrB* gene sequencing and phylogenetic analysis. Our findings show that the *B. stercoris* 92p strain has strong biocontrol ability against *N. musae*, the causal agent of banana leaf spot disease, as well as numerous other fungal pathogens of bananas, including *Cur. lunata*, *P. capitalensis*, and *Nigr. sphaerica*.

In vitro antagonism assays demonstrated that the 92p strain effectively inhibited the mycelial growth of *N. musae*, with an inhibition rate of 76.71%. Moreover, the 92p strain exhibited broad-spectrum antifungal activity, with inhibition rates above 70% against several other major banana fungal pathogens, with the highest being 83.23%. According to Wang et al., *B. stercoris* LJBS06 showed inhibition rates of 50 to 75.05% against *Colletotrichum gloeosporioides*, *Coniothyrium diplodiella*, *Botrytis cinerea,* and *Magnaporthe oryzae* [18]. Another study also reported that the *B. stercoris* B.PNR1 strain showed inhibition rates of 50–70% against pathogenic fungi such as *Sclerotium rolfsii*, *C. musae*, and *C. gloeosporioides* [19]. Compared to previous studies, the 92p strain exhibited higher inhibition rates against banana pathogenic fungi, and its strong antagonistic activity against *N. musae* highlighted its potential as a biocontrol agent for banana leaf spot disease.

Furthermore, microscopic analysis of fungal pathogen hyphae treated with 92p revealed significant morphological alterations, including severe deformities, swelling, and dissolution. The hyphae of *Cur. lunata* and *E. rostratum* exhibited a bead-like appearance at each internode, while the conidia of *Cor. torulosa* and *Neo. clavispora* underwent vacuolation. These findings are consistent with the observations of Mohammed Khadiri et al., who reported that pathogens treated with *B. cereus* B8W8 exhibited various structural changes in the mycelia of the fungal pathogen, including hyphal enlargement, vacuolation, and deformation [29]. Similarly, Yang et al. reported that the mycelia of *F*. *solani* showed incomplete staining, swollen hyphal extremities, increased thickness of the hyphal septa, and ultimately complete fragmentation within a 24-h period after being treated with *B*. *amyloliquefaciens* strains CMS5 and CMR12. In addition, the spores subjected to CMS5 and CMR12 treatments demonstrated deformation and swelling at the ends or middle, resulting in a significant delay in their germination process [30]. These observations indicate that Strain 92p significantly impacts the morphology and growth of fungal pathogens, potentially contributing to its strong antagonistic activity.

To evaluate the ability of *B. stercoris* 92p to control diseases, pot experiments were conducted. In this study, Strain 92p had strong inhibitory activity against *N. musae* in banana leaves, with a biocontrol efficacy of 61.55%. The biocontrol efficacy of Strain 92p is comparable to that reported for other *Bacillus* strains against various plant diseases. For instance, Duan et al. reported that *B. subtilis* JF-4 and *B. amylum* JF-5 demonstrated biological control efficiencies of 48.3% and 40.3%, respectively, against Fusarium wilt in banana in a greenhouse experiment [12]. Similarly, Xie et al. isolated *B. velezensis* YC89 from sugarcane leaves, which significantly inhibited red rot disease caused by *C. falcatum*, achieving a control effect of 61.91% against sugarcane red rot disease [31].

In order to elucidate the biocontrol mechanisms of *B. stercoris* 92p, we conducted a comprehensive investigation of the strain’s genome using whole-genome sequencing. The result revealed that *B. stercoris* 92p harbors genes associated with biocontrol and plant growth promotion, including secondary metabolites (for instance, fengycin, bacillaene, and surfactin), hydrolases (such as amylases and proteases), phosphorus metabolism and genes involved in colonization, swarming, and biofilm formation. The results establish a genetic foundation for the biocontrol and plant growth-promoting activities of the *B. stercoris* 92p.

Biochemical experiments verified that 92p produces protease and amylase. These lytic enzymes reduce plant pathogen growth by disrupting fungal cell walls [32,33]. Lipopeptides produced by *B. stercoris* 92p were investigated by UHPLC-QTOF-MS/MS analysis. In this study, we found that there were two main antifungal active substances in the lipopeptides extraction, fengycin, and surfactin. In order to identify the structure of fengycin and surfactin, MS/MS analysis was performed. According to the typical fragments in secondary mass spectrometry, surfactin was identified as C14 surfactin B and C14–C15 surfactin A. fengycin was assigned to C16-C17 fengycin A and B. The sequence of C14 surfactin B was C14 *β*-hydroxyl fatty acid chain-Glu^1^ -Leu^2^ -Leu^3^ -Val^4^ -Asp^5^ -Leu^6^ -Val^7^, and C14-C15 surfactin A had a peptide sequence of Glu^1^ -Leu^2^ -Leu^3^ -Leu^4^ -Asp^5^ -Leu^6^ -Leu^7^ and a C14-C15 *β*-hydroxyl fatty acid chain. The C16-C17 fengycin A sequences were Glu^1^ -Orn^2^ -Tyr^3^ -Thr^4^ -Glu^5^ -Ala^6^ -Pro^7^ -Gln^8^ -Tyr^9^ -Ile^10^ with a C16-C17 *β*- OH fatty acid. The sequences of C16-C17 fengycin B could be concluded as Glu^1^ -Orn^2^ -Tyr^3^ -Thr^4^ -Glu^5^ -Val^6^ -Pro^7^ -Gln^8^ -Tyr^9^ -Ile^10^ and a C16-C17 *β*- OH fatty acid. Previous studies only reported gene clusters encoding fengycins and surfactins in the whole-genome sequencing and secondary metabolite prediction of *B. stercoris*, but no identification was performed. To our knowledge, this is the first report on *B. stercoris* related fengycins and surfactins. These lipopeptides have been documented to exhibit strong antifungal activity against a range of plant pathogenic fungi. In particular, fengycins are known for their strong fungicidal activity against filamentous fungi, while surfactins inhibit the spread and colonization of fungi by disrupting the growth and development of fungal hyphae [34,35]. The detection of these lipopeptides in the 92p strain further supports its potential as a biocontrol agent for controlling *N. musae*.

Although our findings provide strong evidence for the biocontrol potential of *B. stercoris* Strain 92p against CLS in banana, we should consider that the strain efficacy may be influenced by environmental conditions, such as temperature, humidity, and soil properties. Therefore, further investigation is necessary to assess Strain 92p’s performance under field conditions. The potential impact of *B.stercoris* 92p on non-target organisms, as well as on the leaf and the soil microbial community, must be thoroughly evaluated to ensure its ecological safety. Additionally, Strain 92p should be investigated for compatibility with various disease management techniques, including pesticides, to establish integrated disease management programs.

## 5. Conclusions

In conclusion, our study demonstrates that *B. stercoris* 92p is a promising biocontrol agent against cordana leaf spots in banana caused by *N. musae*. The strong antagonistic activity, broad antifungal spectrum, and effective biocontrol in pot experiments emphasize the capacity of Strain 92p as an environmentally friendly substitute to chemical fungicides. Genomic investigation of Strain 92p revealed that the genes related to biocontrol and plant growth-promoting properties offer a genetic foundation for the observed phenotypic traits. Moreover, the synthesis of lipopeptides by Strain 92p indicates its potential suitability for combating a diverse array of phytopathogenic fungi. Nevertheless, further research is required to enhance the practical implementation of Strain 92p in the field, assess its long-term stability and ecological impact, and develop commercially viable formulations. Our findings contribute to the development of sustainable disease management strategies in banana production and establish a foundation for future investigations into the biocontrol capabilities of *B. stercoris*.

## Figures and Tables

**Table 1:** Analysis of gene clusters of secondary biosynthetic metabolites in *B. stercoris* 92p.

Gene Clusters	Cluster Type	Product	Start-End Position in the Genome	Identity (%)	Source Strain
Cluster 1	T3PKS	-	955,290–995,947	-	-
Cluster 2	terpene	-	1,043,278–1,064,120	-	-
Cluster 3	NRPS	fengycin	1,128,972–1,206,171	100	BGC0001095
Cluster 4	NRPS	bacillaene	1,282,951–1,388,148	100	BGC0001089
Cluster 5	terpene	-	1,981,498–2,002,305	-	-
Cluster 6	T1PKS	zwittermicin A	2,412,315–2,492,924	18	BGC0001059
Cluster 7	NRPS	surfactin	2,759,681–2,822,513	82	BGC0000433
Cluster 8	NRPS	bacillibactin	3,100,621–3,147,758	100	BGC0000309
Cluster 9	sactipeptide	subtilosin A	3,692,153–3,713,765	100	BGC0000602
Cluster 10	other	bacilysin	3,724,192–3,765,611	100	BGC0001184
Cluster 11	RRE-containing	-	3,963,689–3,983,959	-	-

NRPS: nonribosomal peptide synthetases, T1PKS: type I polyketide synthase, T3PKS: type III polyketide synthase, RRE: (rifampin resistance element).

**Table 2:** Mass peaks of surfactin and fengycins from *Bacillus stercoris* 92p.

No.	Mass Peak, *m*/*z*	Assignment	Sequence
1	1008.6574	C14SurfactinB [M + H]^+^	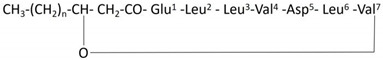
2	1022.6784	C14SurfactinA; [M + H]^+^	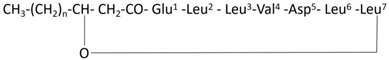
3	1036.5731	C15SurfactinA; [M + H]^+^	
4	732.4121, 1463.8242	C16fengycinA; [M + 2H]^2+^, [M + H]^+^	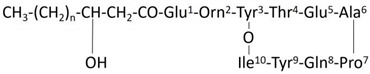
5	739.4112, 1477.8224	C17fengycinA; [M + 2H]^2+^, [M + H]^+^	
6	746.4169, 1491.8338	C16fengycin B; [M + 2H]^2+^, [M + H]^+^	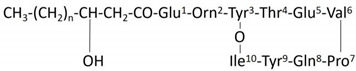
7	753.4319, 1505.8638	C17fengycin B; [M + 2H]^2+^, [M + H]^+^	

## Data Availability

All the data is available in the manuscript.

## Supplementary Materials

The following supporting information can be downloaded at: https://www.mdpi.com/article/10.3390/biom14121495/s1, Figure S1: Colony morphology of strain 92p on LB; Figure S2: COG function classification of strain 92p; Figure S3: GO annotation of strain 92p; Figure S4: KEGG classification of strain 92p; Figure S5: Total ion chromatography of lipopeptides produced by B.stercoris 92p and surfactin standard. (A) lipopeptides of B. stercoris 92p, (B) surfactin standard.; Figure S6: MS/MS spectra of surfactin. (A): *m*/*z* 1008.6653, (B): *m*/*z* 1022.6847, (C): *m*/*z* 1036.6922. Table S1: In vitro antagonistic activity of bacterial strains isolated from banana rhizosphere soil against Neocordana musae.
