# Supplementary material for: Insight into Antifungal Metabolites from Bacillus stercoris 92p Against Banana Cordana Leaf Spot Caused by Neocordana musae
Source: Biomolecules. 2024 Nov 24;14(12):1495. doi: 10.3390/biom14121495 (PMC11672926; doi:10.3390/biom14121495)
Supplement: Supplementary file 1 [file biomolecules-14-01495-s001.zip › biomolecules-3272279-supplementary.pdf]

Insight into antifungal metabolites from *Bacillus stercoris* 92p against banana cordana leaf spot caused by *Neocordana musae*

Qunfang Yu[1,2], Pengbo He[1], Yanxiang Qi[2], Pengfei He[1], Ayesha Ahmed[1], Xin Zhang[2], He Zhang[2], Yixin Wu[1], Shahzad Munir[1]*, Yueqiu He[1]*

Table S1 In vitro antagonistic activity of bacterial strains isolated from banana rhizosphere soil against *Neocordana musae*.

Strains	Inhibition rate（%）	Strains	Inhibition rate（%）
44-1	62.99±4.48	92p	76.71±7.09
49-2	69.25±3.4	93	55.78±2.49
51	58.1±3.06	95	61.77±1.65
70	63.67±1.47	98	72.11±1.43
76	50.88±2.05	107	68.98±3.24
78	62.45±3.08	113	69.12±4.48
81	68.71±4.25	115	69.12±2.46
84	60.14±3.06	129	68.16±2.94
89	67.89±4.48	144	70.48±4.77

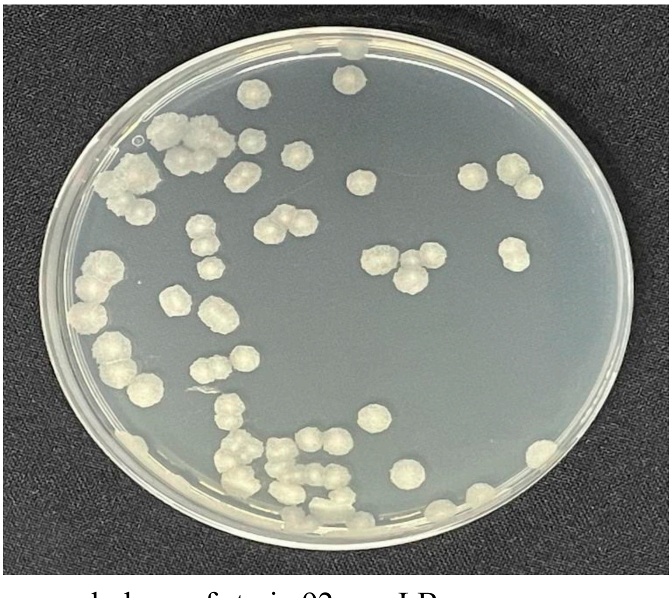

Figure S1 Colony morphology of strain 92p on LB

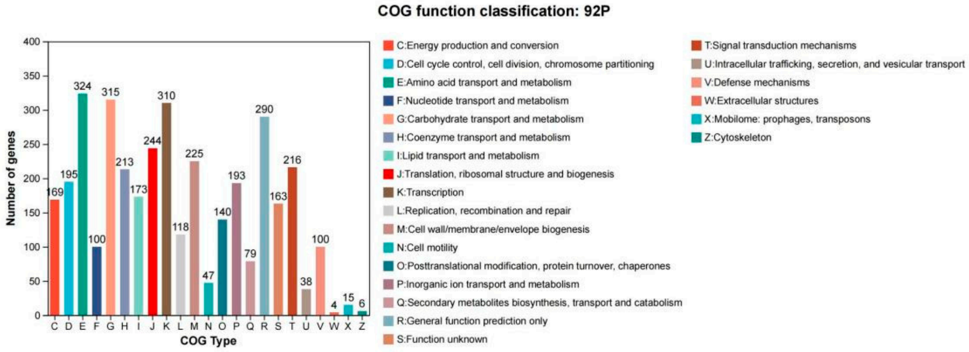

Figure S2 COG function classification of strain 92p

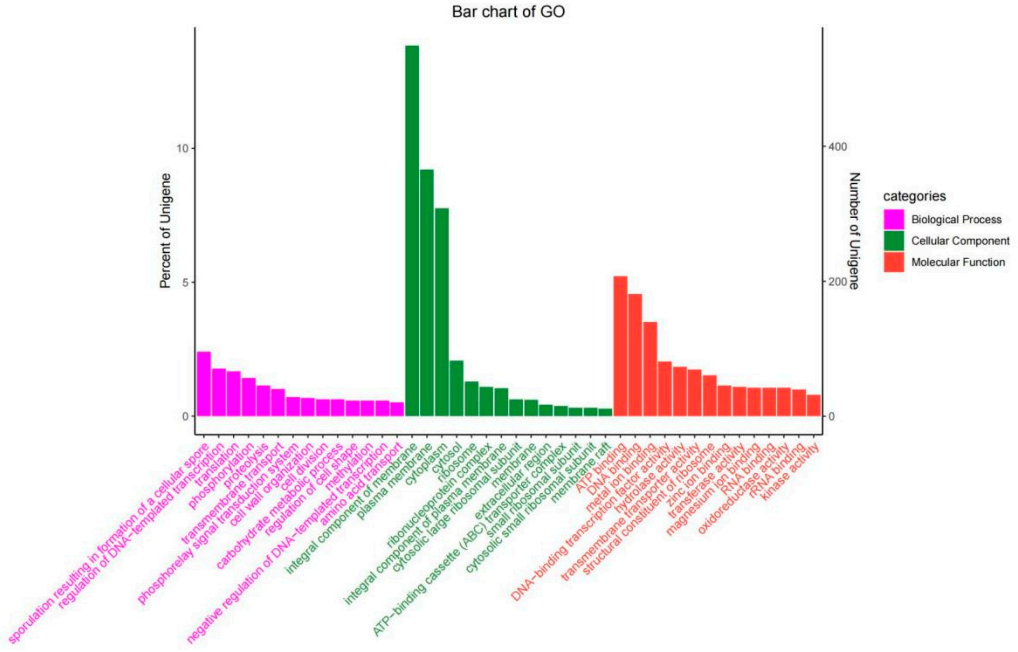

Figure S3 GO annotation of strain 92p

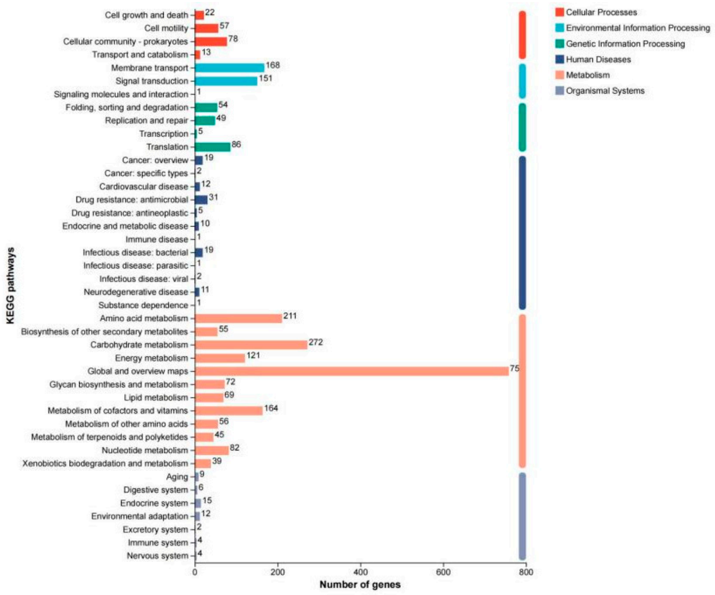

Figure S4 KEGG classification of strain 92p

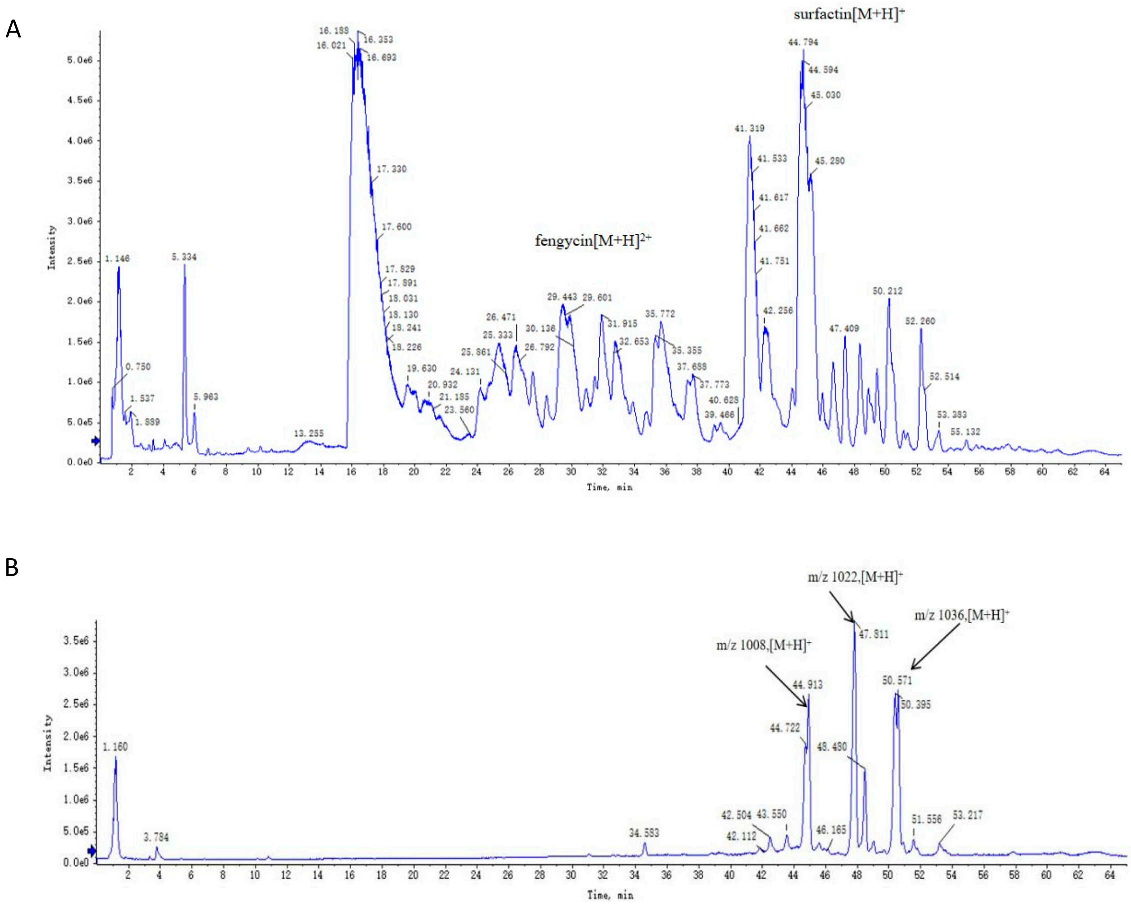

Figure S5 Total ion chromatography of lipopeptides produced by *B.stercoris* 92p and surfactin standard. (A) lipopeptides of *B. stercoris* 92p, (B) surfactin standard.

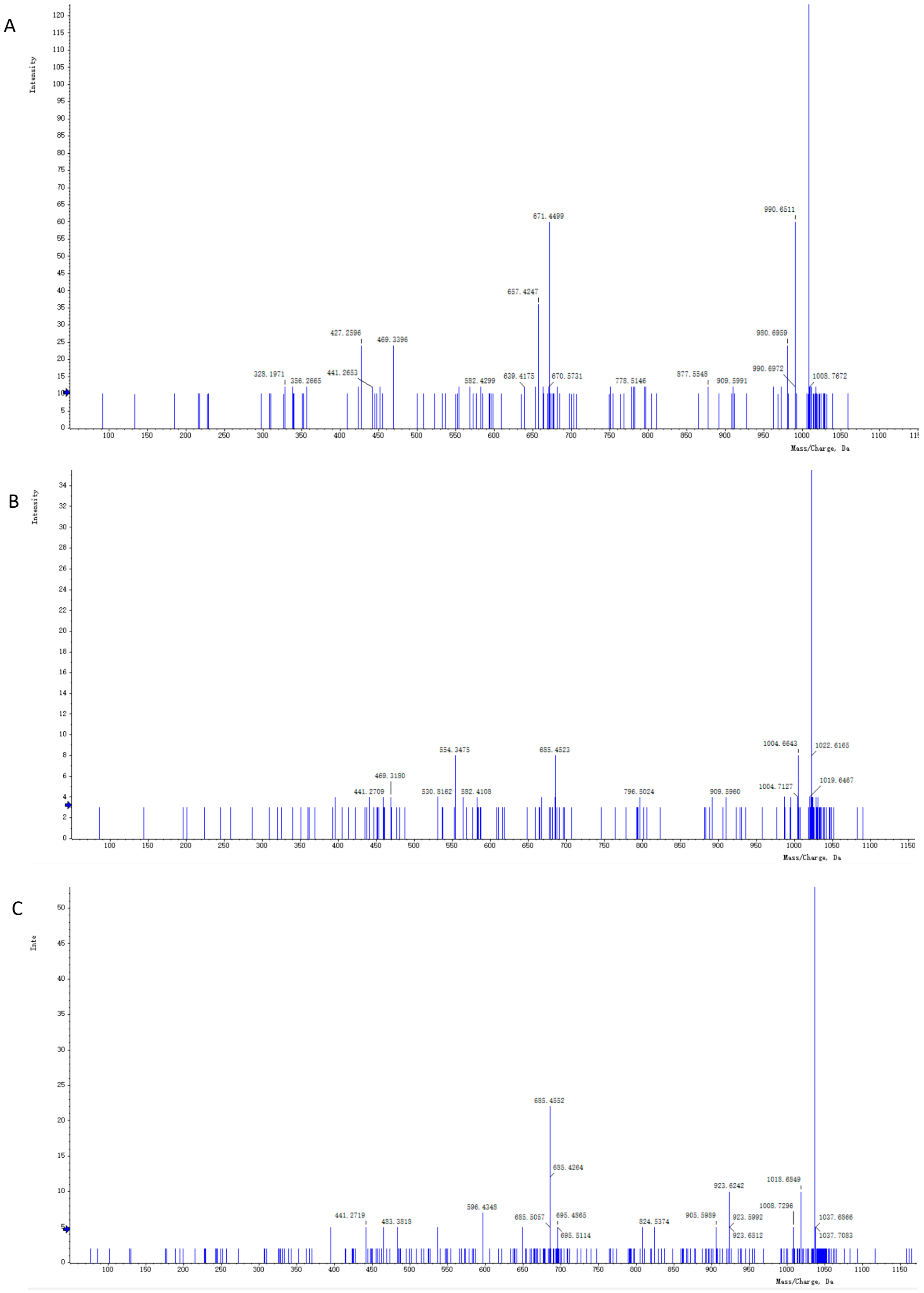

Figure S6 MS/MS spectra of surfactin . (A):*m/z* 1008.6653,(B):*m/z* 1022.6847,(C):*m/z* 1036.6922.
